# A review of adverse events in animals and children after secondary exposure to transdermal hormone‐containing medicinal products

**DOI:** 10.1002/vro2.48

**Published:** 2022-10-28

**Authors:** Karin Sjöström, James Mount, Anna‐Karin Klocker, Veronica Arthurson

**Affiliations:** ^1^ Veterinary Medicine Group Department of Drug Safety Swedish Medical Products Agency (Läkemedelsverket) Uppsala Sweden

## Abstract

**Background:**

Hormonal replacement therapy is widely used to treat conditions in humans, the most well‐known indication being the relief of menopausal symptoms in women. Many of the hormone‐containing products (HCP) are applied to the skin. This transdermal delivery poses a risk to animals and humans through secondary exposure, especially when product information is not strictly followed. The aim of this article is to raise awareness among veterinarians and human healthcare providers of this risk; based on evidence from spontaneous reporting of suspected adverse events (AEs) in animals and humans. Interventions are also explored to mitigate the risk of secondary exposure to transdermal HCP (THCP).

**Review of spontaneously reported suspected AEs:**

The Swedish Medical Products Agency has received several, mainly serious, AE reports in animals and children following secondary exposure to THCPs. The AE reports were reviewed together with worldwide data from the EudraVigilance Veterinary database and human EudraVigilance Data Analysis System. The clinical signs reported in animals included persistent signs of oestrus, poor growth rate and birth defects. In humans, reported clinical signs included precocious puberty, unresolved virilisation, accelerated growth rate and female infertility.

**Conclusions:**

It is important that THCP are used according to manufacturer's instructions and users are made aware of risks and mitigating measures. This review of AEs in animals and children provides evidence to show that the use of THCP poses a risk for secondary exposure. Efficient communication strategies that stretch across veterinary and human medicinal disciplines are required to raise mutual awareness and minimise the risk of AEs in animals and humans.

## Introduction

Hormonal replacement therapy (HRT) is widely used to treat a number of medical conditions in humans. Relief of menopausal symptoms is the most well‐known indication for exogenous oestrogen in women and it has been used since 1942.^1^ Testosterone is frequently used in men with low or no natural production, such as in male hypogonadism or hypoandrogenism. Transdermal products have been used more frequently in the last decade due to fewer complications compared to oral formulations.^1^, ^2^, ^3^

Transdermal administration of oestrogen‐ and testosterone‐containing medicinal products (TOCP and TTCP) on humans can be achieved with skin patches, gels, creams and sprays. The reported advantages of transdermal administration include quicker onset, reduced impact on liver function and reduced risk of venous thromboembolism (VTE).^4^, ^5^, ^6^, ^7^, ^8^ Some disadvantages include inaccurate dosage, difficulties with application and compliance, skin irritation at the site of application and the risk of secondary exposure.^2^, ^4^, ^9^, ^10^ There are no published reports of secondary exposure, including spontaneously reported adverse events (AEs), following the use of transdermal patches. Although, transdermal patches, containing other active substances, have been associated with other types of unintentional exposure, such as ingestion or accidental skin contact.^11^, ^12^ The cases of secondary expose described in this article are limited to gel, cream and spray formulations.

Secondary exogenous exposure of transdermal hormone‐containing products (THCP) poses a risk to both animals and humans.^13^ The risk is significantly increased when users fail to use the product in accordance with the product information. Young animals and children are at greatest risk due to their relatively small size, early stage of development and higher likelihood of physical contact with adults within a home setting.^14^, ^15^, ^16^, ^17^, ^18^, ^19^ The US Food and Drug Administration (FDA) has reported cases of unintentional exposure to oestrogen, both in children and animals, since the first transdermal products were approved and marketed.^13^, ^17^, ^20^ Over the last 10 years, the US FDA has published literature, safety announcements and other publications within the USA in an attempt to highlight the risk of THCP in animals and humans. Despite this increased awareness, cases of secondary exposures to both oestrogen and testosterone are still being reported.^21^

Secondary exposure is also a matter of worldwide concern, although there is limited published information. Oestrogen‐induced alopecia is well described in dogs and has been linked in some cases to exogenous hormonal exposure.^17^, ^22^ It is possible that secondary exposure to a THCP can be overlooked as a differential diagnosis; for example, it was observed in dogs with signs of ovarian remnant syndrome.^19^ To the best of the authors’ knowledge, there has only been one previously documented case of secondary exposure to a TOCP for cats and exogenous exposure to TTCPs in any animal has not been described previously.^23^ In children, there are a relative limited number of published cases of secondary exposure to THCP.^15^, ^18^, ^24^

In recent years, the Swedish Medical Products Agency (SMPA) has received reports of suspected AEs regarding cats, dogs and children who were secondarily exposed to TOCPs or TTCPs. These cases will be reviewed in this article together with complimentary information on worldwide AEs, derived from the EudraVigilance Veterinary (EVV) database and the human EudraVigilance Data Analysis System (EVDAS). These are pharmacovigilance databases of the European Medicines Agency containing reports of suspected AEs in animals and humans. The global definition of an AE is stated in the guidance from the Veterinary International Conference on Harmonization and built on further in the EU Regulation 2019/6.^25^, ^26^ The terms AE and adverse reaction are in some cases used differently between veterinary and human pharmacovigilance activities. However, in this article, the term AE will be used consistently for both disciplines.

The aim of this article is to raise the awareness of the risks of secondary exposure to THCPs in animals and children, based on evidence from reports of spontaneous AEs. In particular, it is important that prescribing human healthcare providers, together with pharmacists, inform patients about the correct use of THCPs and the associated risks. The evidence in this article should also place veterinarians in a better position to recognise potential cases of secondary exposure in animals. This in turn should encourage both veterinarians and human healthcare providers to report such AEs to the relevant competent authority or marketing authorisation holder (MAH). Potential interventions are also explored to mitigate the risk of secondary exposure to THCPs.

## Review of spontaneously reported suspected adverse events in animals

Between October 2019 and May 2022, the SMPA received six reports of suspected AEs in animals, including 15 individual animals, related to secondary exposure to TOCPs; used by animal owners in the home‐setting. Furthermore, a review of AE reports in animals recorded in the EVV database was also performed and summarised below. In the described animal reports, the TOCPs used by animal owners relates to a spray formulation, which delivers 1.53 mg of oestradiol per dose, applied to the forearms. The number and frequency of doses were determined by the prescribing human healthcare providers. The MAH recommends that the lowest dose possible should be used and individual doses should be placed on different parts of the forearm, with up to three sprays per arm. The TOCPs used were nationally authorised medicinal products and were not compounded products. Summaries of the reports in Sweden involving the 15 animals are given below; please note that not all cases had exactly the same information reported on all occasions.

### Report 1 (dog)

**FIGURE 1:**
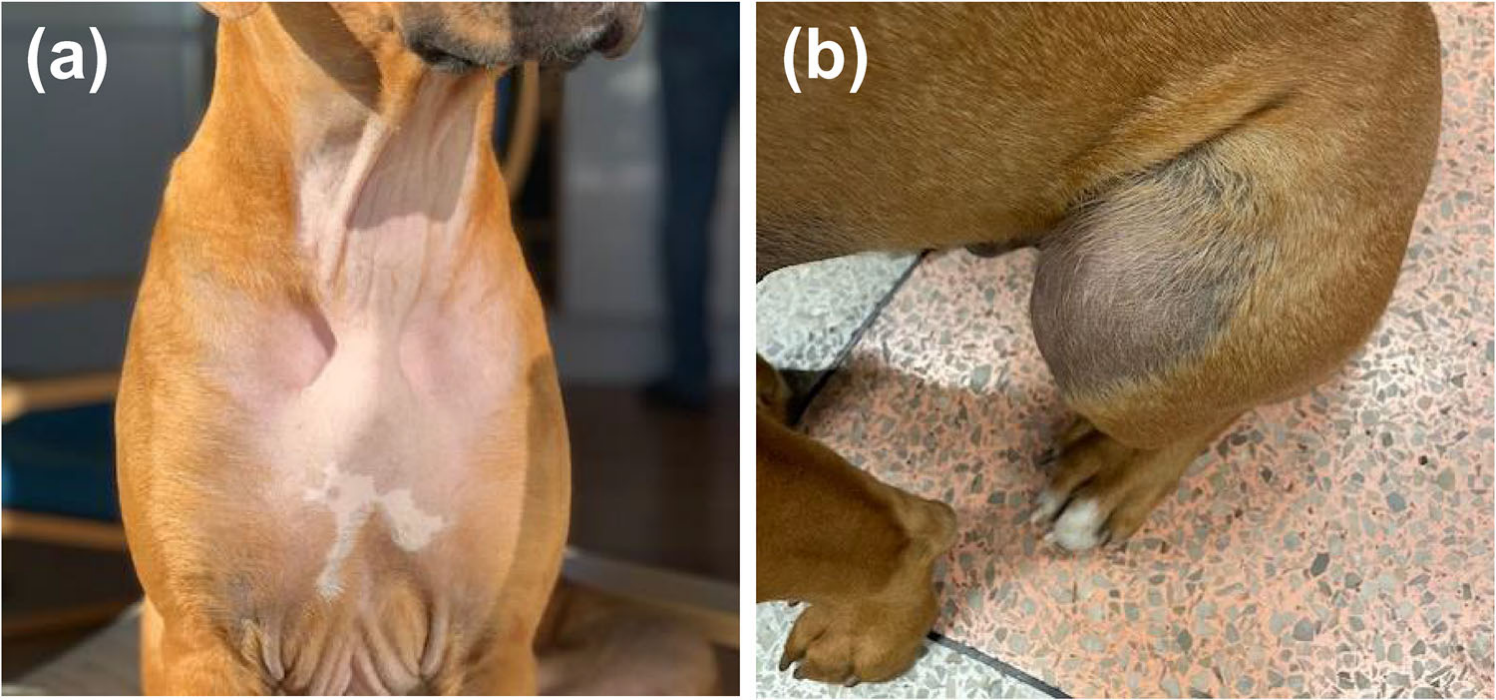
Symmetrical alopecia in a 6‐month‐old, entire male, Rhodesian ridgeback dog following secondary exposure to a transdermal oestrogen‐containing product. (a) Symmetrical alopecia of the pectoral region. (b) Alopecia of the dorsal aspect of thigh. Full details are found in report 1 in the main text.

A female animal owner received HRT during an unspecified period of time. A TOCP in a spray formulation was used. A 6‐month‐old, entire male, Rhodesian Ridgeback dog lived in the household. The dog was introduced to the household at 2 months of age. The dog was presented to a veterinary surgeon after developing symmetrical alopecia (ventral perianal region, dorsal and caudal aspects of thighs and pectoral region) (Figure 1a,b). Clinical examination revealed very small testes with one testis remaining within the inguinal canal, mammary hypertrophy and a so‐called preputial edge; normally seen only in cases with testicular tumours. Cytology performed on a sample from the preputium revealed cellular keratinisation, which is often observed in bitches in oestrus or with hyperoestrogenism. Ultrasonography revealed small testes with a normal structure. Clinical pathological assessment of the blood revealed non‐detectable testosterone level and high oestradiol level, which was considered to be consistent with a bitch in oestrus. Following discussions between the clinician and the animal owner, involvement of the HRT was suspected. The animal owner reported that the puppy had been lifted into the owners bed each evening approximately 5 min after application, using the same arm. Subsequently, the animal owner's treatment was withdrawn and 7 weeks later the dog's hair had started to grow back, the testes increased in size and the mammary glands reduced in size.

### Report 2 (three puppies)

**FIGURE 2:**
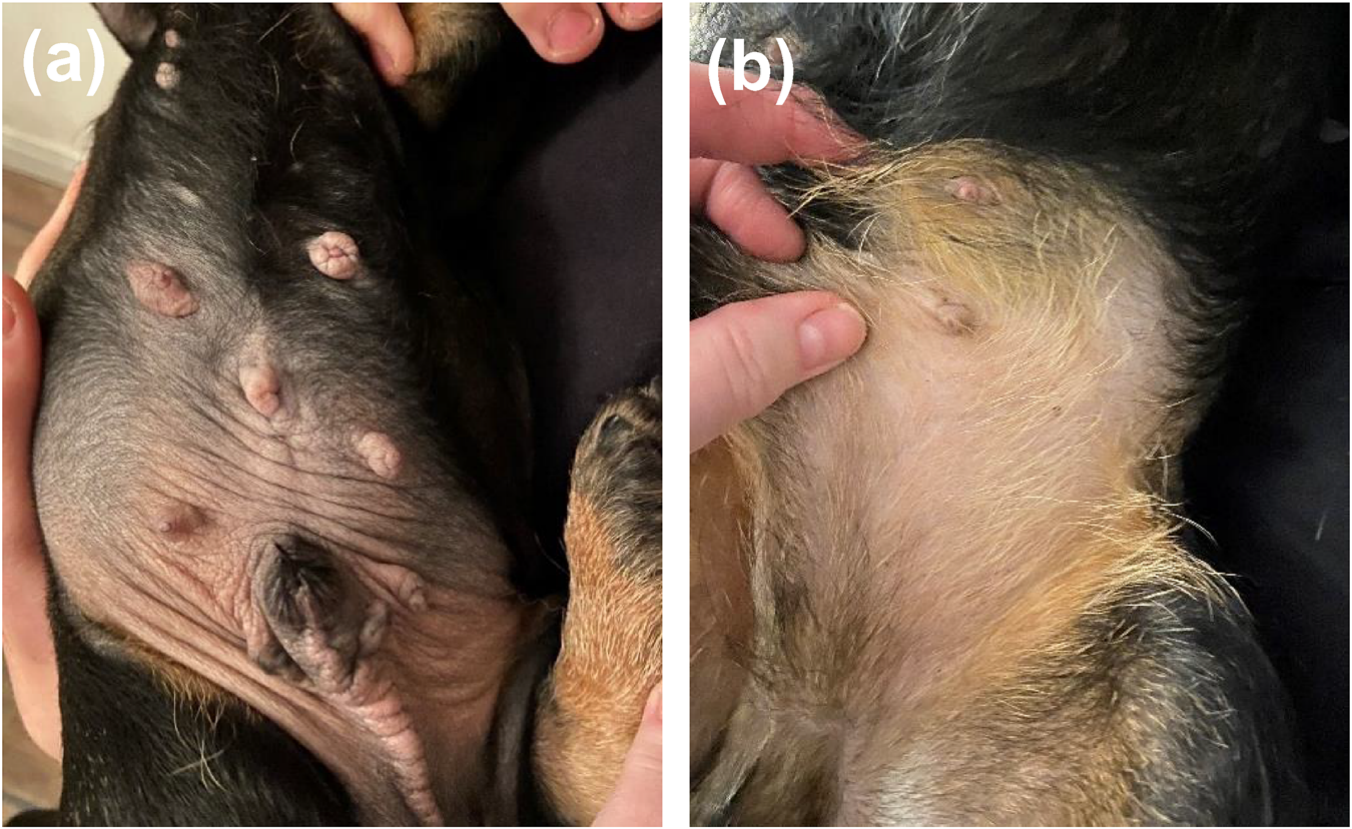
An 8‐week‐old, wire‐haired Dachshund before and after secondary exposure to a transdermal oestrogen‐containing product. (a) Mammary hypertrophy, hypertrophied abdominal skin, hanging preputium and bilateral non‐palpable testes following long‐term secondary exposure to a transdermal oestrogen‐containing product (HRT) which was used by the animal's owner. (b) One year after withdrawal of the HRT in the animal owner. The clinical signs show almost complete resolution. Full details are found in report 2 in the main text.

A female animal owner received HRT for a period of 2 years. A TOCP in a spray formulation was used, with two metered dose‐sprays daily. In the household, three male puppies developed clinical signs suspected to be AEs related to secondary exposure. The three, 8‐week‐old, wire‐haired Dachshund puppies were presented to a veterinary surgeon with mammary hypertrophy, hypertrophied skin and alopecia of the ventral abdomen, hanging preputia and bilateral non‐palpable testes (Figure 2a). Following discussions between the animal owner and the clinician, the clinical signs of the puppies were suspected to be AEs related to the HRT. The animal owner reported that the puppies had been carried on the same arm where the HRT spray had been administered, although the dogs were never carried immediately after administration. The HRT was stopped and 1 year later the puppies had almost completely recovered (Figure 2b).

### Report 3 (three cats)

A female animal owner received HRT to treat menopausal symptoms using a TOCP in a spray formulation; applied to the upper arms with two metered dose‐sprays daily. Three cats in household presented with clinical signs, which were suspected to be AEs related to secondary exposure. Approximately, 2 weeks after the initiation of HRT by the animal owner, a 3.5‐year‐old, neutered female Sphynx cat presented with exaggerated and persistent signs of oestrus (excessive vocalisation, decreased appetite, extra‐affectionate behaviour and raised hindquarters). Clinical examination by a veterinary surgeon revealed a good general health status of the cat despite the clinical signs. Ovarian remnant syndrome was suspected and treatment with deslorelin was initiated. A 2.5‐year‐old, male neutered Sphynx cat, from same household developed clinical signs, although it was not examined. Reported signs included anxiety, increased signs of territorialism and increased sexual interest in the female cat.

A month later, the female cat showed no signs of improvement and the signs progressed to include strong vocalisation, unusual territorial urination outside of the litterbox, hyporexia, insomnia and abnormal ‘psychotic’ behaviours. Gabapentin was administered to the cat. The following month, the female cat was clinically examined, the general health status of the cat had deteriorated despite treatment with gabapentin. Progesterone therapy was administered to the cat. A week later, the exaggerated signs of oestrus persisted and the feline's general health status deteriorated further. Ultrasonography revealed anechoic fluid on the uterine stump compatible with a small remnant of the right ovary. An explorative laparotomy was performed and no ovarian remnants were identified. The animal owner elected to euthanase the cat due to the significant and rapid deterioration of its general health and persistence of the exaggerated signs of oestrus.

**FIGURE 3:**
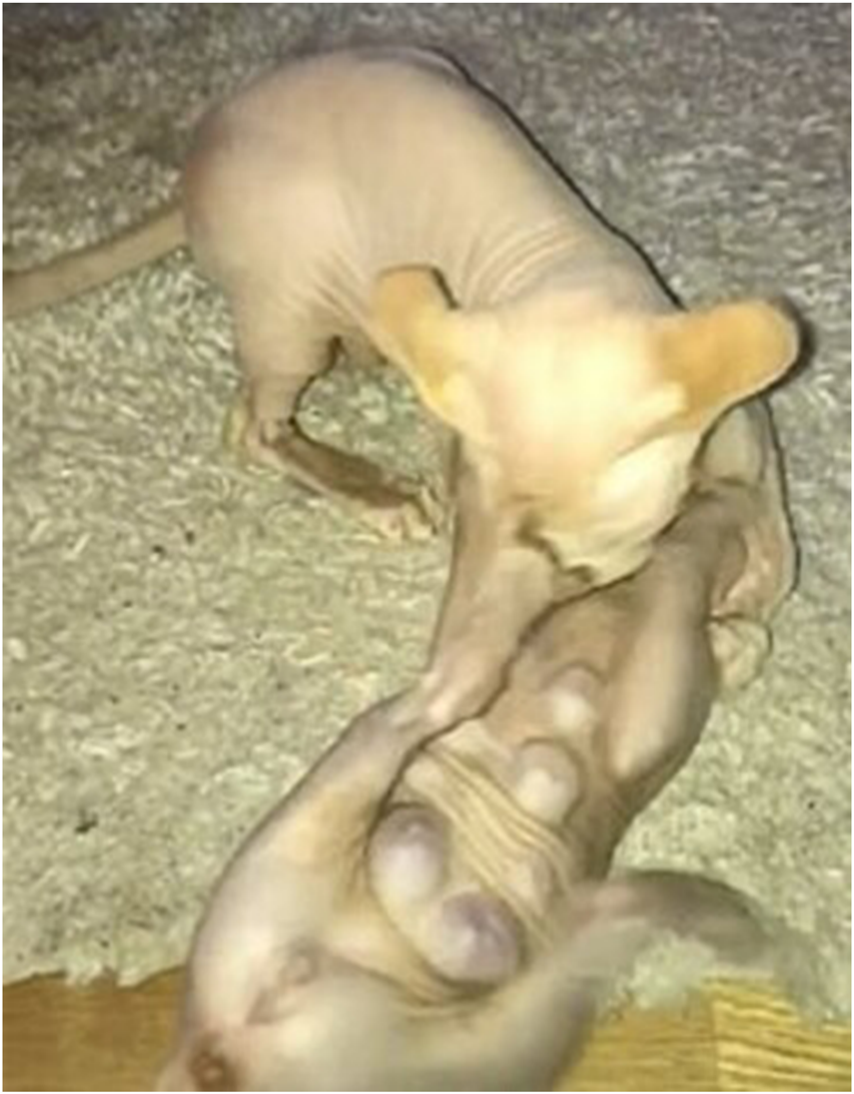
Enlargement of mammary glands in a 12‐week‐old, entire male, Sphynx kitten. The kitten developed clinical signs shortly after introduction into a household where the owner was using a transdermal oestrogen‐containing spray product. Full details are found in report 3 in the main text. The picture is an extracted frame from a video with poor resolution. A segment of the video has been made available as Supporting Information S1.

A 12‐week‐old, Sphynx, entire male kitten was subsequently introduced into the household. Shortly thereafter, the owner observed unusual and significant enlargement of its mammary glands. This was confirmed on examination by a veterinary surgeon, which also revealed ulceration of the caudal mammary glands, unusual microorchidism and sexual immaturity (Figure 3). Following discussions with animal owner, involvement of HRT was suspected as the cause of the AEs and the owner's treatment was subsequently stopped. The male kitten was treated with aglepristone (aglepristone is a competitive progesterone antagonist that has been used to treat various progesterone‐dependent conditions; it has also been used to terminate pregnancy in some species) and within a week of treatment and withdrawal of the owner's HRT, both male cats started to improve and made a full recovery.

### Report 4 (four cats)

A female animal owner received HRT over a period of 4 years. Initially, TOCP patches were used, these were subsequently replaced with a spray, applied two metered dose‐sprays daily. Two, 2‐year‐old, female, domestic short‐haired cats (crossbred with Norwegian forest cat) lived in the household. The cats had been introduced into the home at 12 weeks of age; they had consistently shown a poor growth rate since introduction. The cats remained small in size and weighed between 2 and 3 kg in adulthood; the animal owner had never observed any obvious signs of oestrus in these cats, and the cats did not receive any medical contraceptives. Both cats were outdoor cats and one of them became pregnant and gave birth to one male and one female kitten. According to the animal owner, both kittens had a stunted growth (2 kg at 2 years of age). A year later, the same female cat had a second pregnancy and gave birth to a single stillborn kitten. The other female cat also gave birth to a single stillborn kitten a further 6 months later. The two female cats were later euthanased due to the development of neurological signs, dermal masses all over the body and rapid deterioration of their general health.

Following discussions between the animal owner and the veterinarian, the clinical signs were suspected to be caused by the owner's HRT. The owner was advised to stop the HRT; the owner subsequently observed a significant increased growth rate when the remaining kittens matured as adults.

### Report 5 (two cats)

Over a period of two years, a female animal owner used a TOCP; a spray formulation which was applied two metered dose‐sprays daily. A 1.5‐year‐old, entire female, domestic short‐haired cat lived in the household as an outdoor cat and did not receive any medical contraceptives. The cat was introduced to the household at 12 weeks of age. Following introduction the cat failed to grow at a normal rate. The cat was small in stature and weighed only 2 kg in adulthood. The cat later gave birth to a kitten with myelocele and malformed legs. The kitten was paralysed and subsequently euthanased. Following discussions between a veterinary surgeon and the animal owner, involvement of the HRT was suspected and the HRT was stopped. The animal owner later reported that the feline's condition improved and started to gain weight.

### Report 6 (two cats)

A female animal owner of two, 7‐year‐old, neutered female, domestic short‐haired cats, was receiving HRT using a TOCP; a spray formulation. Shortly after introduction of the two cats to the household, the smaller of the cats started to show exaggerated signs of oestrus (strong vocalisation, restlessness, extra‐affectionate behaviour and raised hindquarters). The other cat was overweight and did not express any unusual clinical signs. According to the animal owner, the other cat had limited contact with humans, which may explain the lack of clinical signs. Advice was sort from a veterinary surgeon who asked the animal owner about ongoing treatments and suspected that the HRT was the most likely cause of the AE in the smaller cat. The animal owner changed the route of administration of HRT; from a spray to patches. Approximately, 2 weeks later, the clinical signs of the smaller cat progressively diminished. A year later, at springtime, no exaggerated signs of oestrus had reappeared.

### Animal reports in Europe and worldwide

Following a cumulative review of AE reports in animals recorded in the EVV database and in addition to the reports given above from Sweden, two additional reports were identified, involving eight dogs; these are summarised below.

### Report from Germany (2020) regarding seven dogs

A female breeder of French Bulldog and Chihuahua dogs had been using a TOCP spray for approximately 1 year. The product was applied once daily to her forearm to alleviate menopausal symptoms. Between 5 and 8 months after the start of treatment, a total of 11 puppies were born to four adult bitches on the premises. At the time of birth, three female puppies showed enlarged vulvae and one male pup showed enlarged mammary glands. At an unspecified time point, enlarged mammary glands were also observed in some of the four adult bitches. Subsequently, the animal owner changed to an oral route of administration of oestradiol and, since then, the clinical signs in the dogs showed progressive resolution.

### Report from Portugal (2022) regarding one dog

The female owner of a 2‐year‐old, neutered female, miniature Dachshund was undergoing treatment with a TOCP for menopausal symptoms, which was applied to the forearm. Following the start of treatment, the owner observed the following clinical signs in the dog; oedema of the vulva, attraction to male dogs, enlarged mammary glands and bilateral, symmetrical alopecia of the muzzle. Following clinical examination by a veterinary surgeon, ovarian remnant syndrome was suspected, although ultrasonographic examination did not show ovarian remnants. Following a detailed conversation with the animal owner, a connection with the TOCP was established. It was reported that the dog liked to sit on the owner's lap for long periods of time. The veterinarian asked the animal owner to talk with her gynaecologist, who subsequently stopped the HRT. Following withdrawal, the clinical signs in the dog resolved. Interestingly, the owner stated that she had reported this case having read a press release from the General Directorate of Food and Veterinary Medicine in Portugal, which had highlighted the risk of secondary exposure of animals to THCPs.

## Review of spontaneously reported suspected adverse events in humans

In addition to the reports of secondary exposure in animals, the SMPA received one report of a serious AE in a young child presumed to be related to secondary exposure to a TTCP, which is described below.

### Human report in Sweden

An adult male was applying a gel formulation of a TTCP to the skin and the area was consistently covered with clothing. A 14‐month‐old female was presented to a human healthcare provider, at a hospital in Sweden, with a history of having developed signs of virilisation including clitoromegaly, acne and increased body and genital hair. Following investigation, the clinical signs were associated with secondary exposure to the TTCP. Insufficient hand hygiene in the adult male following application of the product was considered as the likely source of the exposure. The adult male expressed that he had been inadequately informed about the risk of secondary exposure to the child via skin contact. At the time of reporting, the clinical signs remained unresolved in the young child following withdrawal of the product. Unfortunately, no further update or information has been received.

### Human reports in Europe and worldwide

A review of AE reports recorded in EVDAS was performed and retrieved relevant reports that had occurred in children both within Europe and worldwide. The reports have been collated, tabulated and divided into two sections based on the active substance including oestrogen and testosterone. In some reports, Tanner Staging, also known as Sexual Maturity Rating, is mentioned, which is an objective classification system to document and track the development of secondary sex characteristics of children during puberty.^27^, ^28^

### Adverse events in children related to secondary exposure to oestrogen‐containing medicinal products

Table 1 shows the details of AEs reported between 2018 and 2021, in children following secondary exposure to TOCPs. A total of 10 reports in children were retrieved. Seven of these reports were classified as serious. The reports involved eight males and two females, with an age range of 3–14 years (median 5 years). Most of the suspected AEs were related to skin exposure to oestrogen, mainly oestradiol, in a spray, cream or gel formulation, following use by a close family member.

**TABLE 1:** Suspected adverse events (AEs) following secondary exposure of children to transdermal oestrogen‐containing medicinal products

Country	Year	Age (years)	Sex	Clinical signs
Denmark	2021	2	**♀**	Gynaecomastia and vaginal discharge
Germany	2019	4.4	**♂**	Serious pseudoprecocious puberty, breast development (Tanner stage 2), accelerated growth rate, premature and advanced thelarche and gender‐related references, although serum gonadotropins were low and appropriate for age
Germany	2019	4.9	**♀**	Pseudoprecocious puberty, breast development (Tanner stage 2), accelerated growth rate and advanced bone age by 2 years
Germany	2020	4.4	**♂**	Pseudoprecocious puberty, breast development (Tanner stage 2), accelerated growth rate, premature and advanced thelarche and gender‐related references
Germany	2021	7	**♂**	Somatomegaly, severely gigantism, gynaecomastia, premature pubarche, precocious puberty, female pseudo puberty (pseudoprecocious puberty), high estradiol level and slight developmental delay
Germany	2021	5	**♂**	Early signs of puberty and body odour
Germany	2021	3	**♂**	Early signs of puberty, significant increased oestrogen levels and premature thelarche
Germany	2020	14	**♂**	Twin brothers developed nipple tenderness and small breast lumps
Germany	2020	14	**♂**	
Spain	2018	3	**♂**	Bilateral gynaecomastia

*Note*: A representative sample of AE reports were retrieved from the EudraVigilance Data Analysis System—the pharmacovigilance database at the European Medicines Agency containing reports of suspected AEs in humans.

### Adverse events in children related to secondary exposure to testosterone‐containing medicinal products

Table 2 shows the details of AEs reported between 2007 and 2020, in children following secondary exposure to TTCPs. A total of 21 reports in children were retrieved. Eight of these reports were classified as serious. The reports involved 10 males and 11 females, with an age range of 6 months up to 7 years (median 3 years). Most of the AE reports were related to secondary exposure to testosterone in a gel form following use by a close family member. It is noteworthy to mention that surgical management was necessary in one report of clitoral enlargement. In another report, a mother within the same household was also affected, with clinical signs of infertility and amenorrhoea.

**TABLE 2:** Suspected adverse events (AEs) following secondary exposure of children to transdermal testosterone‐containing medicinal products

Country	Year	Age (years)	Sex	Clinical signs
Australia	2011	2	♀	Progressive virilisation
Australia	2013	2	♀	Androgenisation (virilism), hair growth abnormal and clitoromegaly
Belgium	2012	5	♂	Central precocious puberty
Brazil	2019	2.6	♀	Clitoromegaly, virilisation, premature development pubic hair (Tanner stage 3) and high testosterone levels
Brazil	2019	1.7	♀	Premature pubarche, tall height, clitoromegaly and virilisation
Brazil	2019	6.8	♂	Severe penis enlargement and virilisation
Germany	2020	0.8	♀	Pseudoprecocious puberty, hyperpigmentation of labia majora, acne, premature body hair and accelerated growth rate
Germany	2020	0.5	♀	Serious pseudoprecocious puberty, hyperpigmentation of labia majora, premature pubarche, accelerated growth rate and advanced bone age by 6 months
Ireland	2009	3	♀	Hyperandrogenism, precocious puberty, premature development of pubic hair (Tanner stage 3), advanced height, no breast development, clitoromegaly and elevated testosterone levels
Spain	2007	2.5	♀	Virilisation (clitoral lengthening)
Spain	2017	6	♂	Hyperandrogenism, premature pubarche, penile growth, advanced height, large testicular volume, elevated testosterone levels, advanced growth rate and advanced bone age by 1.5 years
Spain	2017	2	♂	Hypergonadism and advanced bone age by 3 years
Spain	2017	6.3	♂	Hypergonadism, premature prepuberty and unresolved penis disorder
Spain	2018	2	♂	Penile growth with frequent erections, pubarche, increased testosterone levels and excessive increase in height
Spain	2016	3.7	♂	Scrotal disorder, increased blood testosterone, pseudoprecocious puberty, dysphonia and advanced bone age
Spain	2017	6	♂	Hyperandrogenism, high growth rate and penile growth
Spain	2012	1.5	♂	Precocious puberty (the mother of this child also suffered from long‐term hirsutism, infertility and amenorrhea)
Sweden	2019	1.2	♀	Virilisation, clitoral hypertrophy and premature genital hair
Switzerland	2018	2.5	♀	Extreme elevated testosterone, clitoromegaly, advanced bone age by 2 years, abnormal behaviour, seborrhoea, pubic hair growth and acne
The Netherlands	2015	1.1	♀	Clitoromegaly
UK	2020	7	♂	Aggression (alternating mood between calm and very aggressive)

*Note*: A representative sample of AE reports were retrieved from the EudraVigilance Data Analysis System—the pharmacovigilance database at the European Medicines Agency containing reports of suspected AEs in humans.

## Discussion

The AEs described in this article demonstrate that secondary exposure to THCPs poses a significant risk and concern for animals and humans; with a wide range of undesirable clinical signs and significant consequences in animals and humans. Clinical signs of hyperoestrogenism may range from alopecia and/or prolonged signs of oestrus in neutered cats (both females and males)^17^, ^22^, ^29^, ^30^ to more severe signs including hepatotoxicity, bone marrow suppression,^31^, ^32^ stillbirth and malformations in new‐born animals and humans.^33^, ^34^, ^35^, ^36^, ^37^ Secondary exposure in animals in the reviewed reports of suspected AEs might have led, in some cases, to unnecessary surgical intervention and even avoidable euthanasia of four animals.

In the majority of the reports from Sweden, the AEs in the exposed animals (10 of 15) resolved following withdrawal of the THCP (a positive de‐challenge) and altered route of administration. This suggests a strong causal association between the THCP and clinical signs in these animals. It is important that veterinary surgeons collect a detailed anamnesis in order to obtain relevant information to facilitate detection of such cases. Secondary exposure to THCP should be considered as a differential diagnosis in cases of unexplained clinical signs.

It is noteworthy to mention that several veterinary surgeons in Sweden have contacted SMPA to indicate their concerns for an increasing frequency of clinical cases related to secondary exposure to THCP. Many of these cases have yet to be formally reported as suspected AEs to the SMPA. Most of these cases relate to dogs and cats that have developed alopecia as a result of hyperoestrogenism. Veterinary surgeons have also observed a rising number of cases discussed between animal owners within social media channels. Veterinarians have considered whether use of THCP has increased, or whether the precautions taken by users have been reduced. Sales of THCPs within Sweden have shown a steady and rapid increase over the last 4–5 years. Based on sales figures published by the Swedish eHealth Agency, the use of TTCPs and TOCPs in Sweden has increased by approximately 31% and 100%, respectively, which could partly explain the recent increase in observed AEs following secondary exposure. Veterinary surgeons in Sweden have expressed to SMPA a need to raise awareness among animal owners of the risk to their pets and even to children, when using THCPs. Swedish veterinarians have expressed, through email correspondence to SMPA or posts in veterinary forums on social media, a need for prescribing human healthcare providers, including pharmacists, to routinely inform users about the importance of correct use of THCPs and the potential risks of secondary exposure.

Cases of secondary exposure of THCPs have been previously documented, both in humans and animals, albeit in small numbers.^18^, ^21^, ^24^, ^38^, ^39^ In dogs, oestrogen‐induced alopecia is well described in the literature and has been previously associated with exogenous hormonal exposure.^17^, ^19^, ^22^, ^39^ Dogs have even been reported to develop physical signs of feminisation in some cases.^17^, ^22^ Prolonged periods of oestrus, observed in the reviewed AE reports, have also been previously documented in a Chihuahua, which developed stump pyometra.^39^

To the best of the authors’ knowledge, there has only been one previously documented case of secondary exposure to a TOCP in cats.^23^ The evidence from the reviewed AE reports further indicates that the risk and consequences are clinically relevant for cats. Furthermore, there are no documented cases of secondary exposure to TTCPs in animals; these could potentially give rise to clinical signs such as aggression or unexplained Cushing's syndrome.

Secondary exposure to a THCP, in some cases, can be overlooked as a differential diagnosis, both in animals and children. This has been observed previously in dogs with signs of ovarian remnant syndrome.^19^ This is also exemplified in report 3, where the establishment of an association with the THCP was delayed and the cat was euthanased due to a deterioration of health status. The secondary exposure to a THCP can, in some human cases, delay the diagnosis of other clinical disease. In a previous report, this was exemplified by the delayed diagnosis of a metastatic adrenocortical carcinoma in an 8‐month‐old child. Initially, the clinical signs in the child had been associated with secondary exposure to a TTCP being used by her father. The clinical signs persisted despite discontinuation of the TTCP and an adrenocortical carcinoma was subsequently diagnosed.^40^

It is important to recognise that secondary exposure to THCPs can have wider impacts within households. In one report, which was retrieved from EVDAS, two young boys were virilised following exposure to the father's TTCP. However, it was later recognised that amenorrhea, abortion and infertility, experienced by the mother, were also related to secondary exposure to the TTCP. Previous publications have reported cases of severe birth defects, low birth weight, toxicity and even tumour development in humans following the exposure to exogenous hormones, including oestrogen, progesterone and testosterone.^33^, ^34^, ^35^, ^36^, ^37^, ^41^, ^42^ This supports further the importance of including relevant questions in the anamnesis of animals cases and human patients when hormonal influences/imbalances or other unclear cases are suspected.

Typically, exposure of animals and children is through the contact with THCP that are applied to the skin of the forearms, which is a common recommended site of application stated in the product information. It should be noted that the risk of secondary exposure is low if appropriate mitigating measures are strictly followed by users as stated in product information. These can include allowing the area to dry and covering the application site with clothing. It has been observed experimentally that no significant transfer of oestradiol occurred following skin‐to‐skin contact between adults, 1 h after application of a TOCP in a spray formulation.^43^ Nevertheless, secondary exposure does occur on rare occasions and young animals and children appear to be at greatest risk as they are increasingly carried in the arms, thus coming into direct contact with the active substance, either absorbed through the skin or potentially ingested. Young animals and children are also more susceptible due to their small body size, early stage of development and higher degree of physical contact with adults within a home setting.^14^, ^15^, ^16^, ^17^, ^18^, ^21^, ^22^, ^24^ It is interesting that some animal owners in the reviewed AE reports from Sweden mentioned that extensive perspiration, a common menopausal symptom, had caused the THCP to run down their arms and hands. This could potentially augment the risk of secondary exposure.

Raising awareness of the risk of secondary exposure to THCPs is a key part of any strategy to reduce the number of animals and children that are affected. The FDA in the USA has made attempts to raise awareness among veterinarians and human healthcare providers through various forms of communication. In one article, the FDA has highlighted the number of AE reports that had been received during the first 3 years after authorisation of the first oestradiol transdermal spray.^13^ This included eight AE reports in children where clinical signs remained unresolved and two AE reports of neutered female dogs that developed mammary enlargement, liver failure and vaginal prolapse. Despite communication activities by the FDA, awareness among veterinarians and human healthcare providers remains low. Some national competent authorities within Europe have taken the initiative to raise awareness at a national level; the animal AE report in this article from Portugal shows that these initiatives can, at least, have a positive effect on reporting rates. Further strategies need to be implemented worldwide to raise awareness, in particular among human healthcare providers when THCPs are prescribed.

Overall, the number of spontaneous AE reports related to secondary exposure to THCPs in animals and children remain low, even cumulatively. As stated in the introduction, cases of secondary expose are limited to gel, cream or spray formulations; the risk is low if these formulations of THCPs are used correctly. Nevertheless, when secondary exposure occurs, the impact on individuals can be significant. It is difficult to determine the true incidence of events within a population through spontaneous reporting alone. Lack of awareness and failure to recognise cases of secondary exposure in both animals and humans could be a possible explanation for the relatively small numbers of AE reports. A low reporting rate in animals could also be a consequence of the previous EU Directive, which stipulated only voluntary reporting of AEs in animals following the use of or exposure to human medicinal products. The EU Regulation, which came into force on 28 January 2022, requires the reporting of AEs related to the use of or exposure to human medicinal products in animals.^26^ It is vital that veterinarians, human healthcare providers and animal owners report these types of AEs to their respective national competent authority to facilitate monitoring. Despite legislative amendments, MAHs of human medicinal products continue to have no obligation to monitor AEs that occur in animals, which could further impact on reporting rates. Despite the low level of reporting, cases of secondary exposure in animals still emerge from other sources such as social media channels. One example is the Veterinary Information Network in USA who, through a review of online message boards, compiled over 100 cases of secondary exposure of animal to THCPs between 2003 and 2011.^44^

Through an analysis of the reviewed AE reports, it is apparent that there seems to be a lack of clear and obvious information provided to users when THCPs are prescribed. It is important to note that most THCPs are accompanied with detailed instruction of use which often include recommended sites of application, the allowance of appropriate drying time before physical contact, use of clothing to cover the application site and other precautions. Human healthcare providers and pharmacists should be encouraged to guide users to this information and make users fully aware of the risks. The risk for secondary exposure in animals and children is low if users adhere to manufacturer's instructions. However, a review, conducted by the authors, of product information which accompanies THCPs, has also revealed that some products do not contain sufficient or relevant warnings to prescribing human healthcare providers or users. This information is required by human healthcare providers to fully inform users when animals and young children are found in the same household. Product information should, at least, cover the risks and the clinical signs which can be expected in individuals, both in animals and humans, when secondary exposure is suspected. It is considered prudent to address this deficiency of product information by encouraging MAHs to harmonising warnings in product information. Apart from highlighting the risk to animals and children, the product information should contain guidance on the management of cases when secondary exposure is strongly suspected.

The risk for secondary exposure should be low when users have received and adhered to guidance in the product information. However, in some situation, additional mitigating measures may need to be considered. There are various options available to help limit secondary exposure. Initially, stopping the THCP could be considered, where appropriate; alternative routes of administration could be explored such as oral dosing. However, oral administration may have higher risk of VTE, pulmonary embolism and deep venous thrombosis compared to transdermal administration.^8^, ^45^ Another alternative could be to explore the possibility to apply THCPs to an alternate area of the body, which is less likely to come into direct contact with animals and children and can be more readily covered with clothing.

One study identified a risk of transferring testosterone by clothing,^46^ and another highlighted a risk of transferring topical products via bedding,^47^ which adds a further complexity to the issue. Suitable alternative application sites would be the lower part of the belly, the groin or upper part of the thighs of human patients, which are already recommended in the product information for some THCP. The MAHs of relevant products should be encouraged to investigate alternative sites for application and potentially harmonise the information related to alternative application site in the product information. Another alternative could be to prescribe transdermal patches to patients with animals or children that are at risk for secondary exposure. As mentioned earlier, secondary exposure to THCP has not been associated with the use of transdermal patches, but it is essential to handle and dispose of patches correctly following use.^11^, ^12^ Routine hand hygiene is also important to emphasise in product information following application of THCP. When measures are taken by the user, evidence from the reviewed AE reports indicates that clinical signs of most exposed individuals, both animals and children, can resolve.

The risks to animals and children related to secondary exposure of THCPs detected via spontaneous AE reporting performed by veterinarians, human healthcare providers and animal owners, demonstrates the importance of pharmacovigilance activities. It also highlights the importance of continuous monitoring of the benefits and risks associated with medicinal products. It is important to encourage reporting of suspected AEs, since reporting of suspected AEs is fundamental to identify and minimise the risks of using veterinary or human medicinal products.^48^, ^49^ Often, some AEs are only detected when a medicinal product has been used by a large number of diverse individuals over a long period of time.

The SMPA is in a unique position as both veterinary and human pharmacovigilance activities are conducted within the same national competent authority. This facilitates the collaboration and crossover between veterinary and human pharmacovigilance activities within Sweden. Evidence presented in this article indicates that there is the need for establishing closer collaboration between human and veterinary pharmacovigilance activities even at a European level. The number of AEs received by the SMPA related to secondary exposure are limited and this could be correlated with the generalised observation of under‐reporting. Under‐reporting has been estimated to be up to 90% in both veterinary and human medicine.^48^, ^50^, ^51^ Numerous factors have been identified that influence human healthcare providers’ motivation or ability to report AEs.^50^, ^51^ Lack of time and access to suitable digital reporting tools have been identified as the most significant barriers for veterinary professionals within Sweden and the UK.^49^, ^52^ Enabling the ability to report AEs via Practice Management Systems has been a recent focus of the SMPA. A solution has been launched for human healthcare providers within Sweden and developments are currently ongoing to implement a similar solution for veterinary professionals in Sweden.

This review of AE reports in animals and humans, together with information gathered from published literature, provides evidence to suggest that the use of THCP in households poses a significant risk of exogenous exposure. We present further evidence that secondary exposure is also relevant in cats. Veterinarians and human healthcare providers should include secondary exposure to THCP as a differential diagnosis when confronted with cases of unexplained clinical signs, or clinical signs indicating a hormonal influence/imbalance. Prescribing human healthcare providers, together with pharmacists, should ensure that users of THCPs are made fully aware of product information and the risks to animals and humans. It is important that THCPs are used strictly according to manufacturer's instructions to minimise the risk of secondary expose. The MAHs of THCPs should be encouraged to investigate additional mitigating measures to further limit risks and harmonise product information. It is crucial that all AEs are reported to the relevant competent authority in order to identify issues and implement necessary measures in a timely manner. This article highlights the need for extension of the one health approach to other areas beyond infectious disease, which could help accelerate the detection of AEs that occur in animals and humans. It is also evident that efficient communication strategies that stretch across global veterinary and human medicinal disciplines are required to raise mutual awareness and minimise the risk of AEs from any medicinal product used in animals and/or humans.

## AUTHOR CONTRIBUTIONS

All authors fulfil the criteria set out in the ICMJE guidelines for authorship and were involved in the writing of the final manuscript. The first draft of the manuscript was written by Karin Sjöström and James Mount with significant editing, revision and contributions from Anna‐Karin Klocker and Veronica Arthurson. Karin Sjöström, James Mount and Anna‐Karin Klocker were involved in data retrieval and review of the adverse events, which are described in the final manuscript. All authors certify that they have participated sufficiently in the work to take public responsibility for the content.

## CONFLICTS OF INTEREST

The authors declare they have no conflicts of interest.

## FUNDING INFORMATION

The authors received no financial support for the authorship and publication of this article.

## ETHICS STATEMENT

The authors confirm that the ethical policies of the journal, as noted on the journal's author guidelines page, have been adhered to. No ethical approval was required.

## Supporting information


**Supporting Information S1**. A video of a 12‐week‐old, entire male, Sphynx kitten. In the video, the 12‐week‐old kitten is seen playing with another cat. Significant enlargement of the mammary glands can be seen, which is a consequence of secondary exposure to a transdermal oestrogen‐containing spray product used by the animal owner. Full details are found in report 3 and a still image from the video is found in Figure 3. The video is in MPEG format.

## Data Availability

Full data sets available upon request to the corresponding author.
